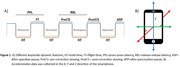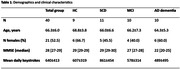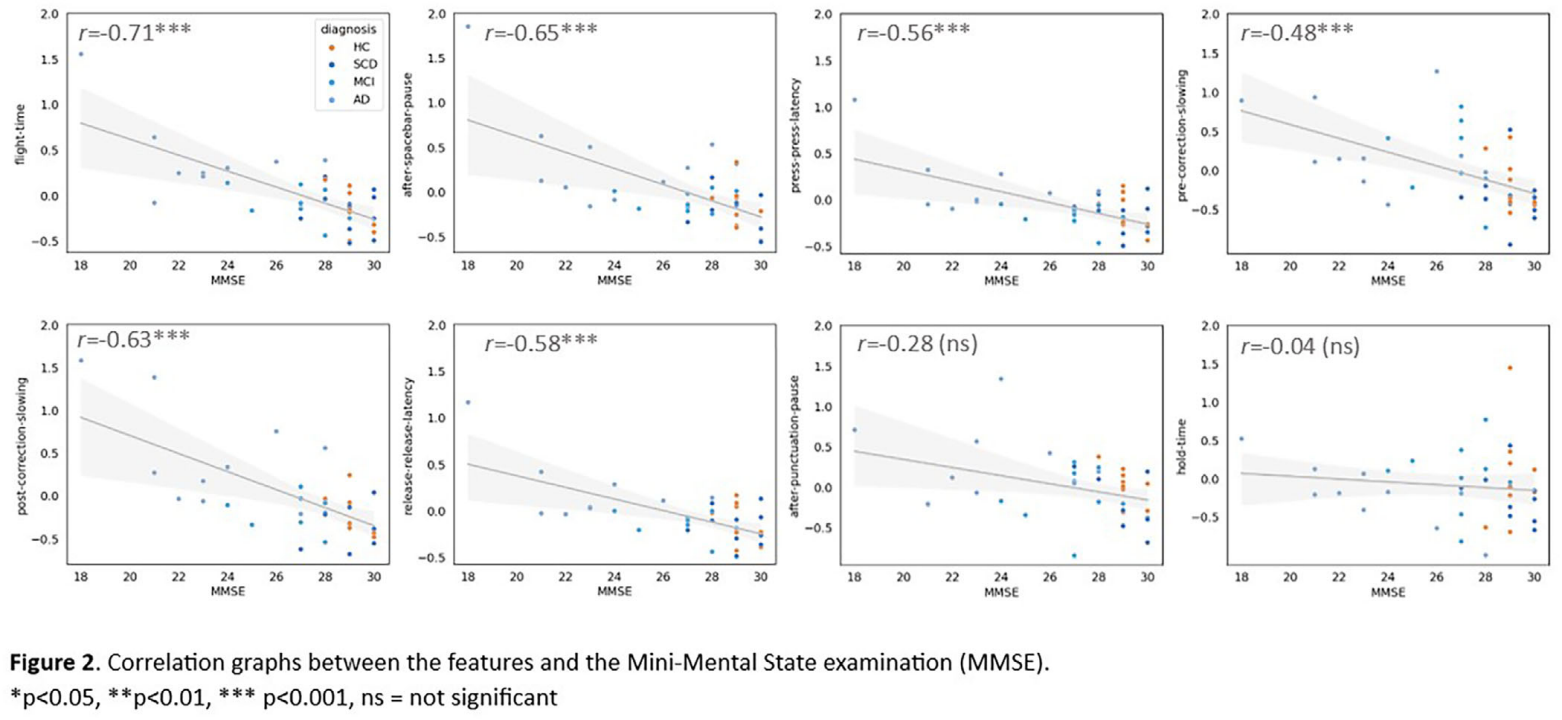# Passively collected smartphone behaviour as a reliable and feasible measure for global cognition in Alzheimer’s Disease

**DOI:** 10.1002/alz.086452

**Published:** 2025-01-03

**Authors:** Matthijs J. Keijzer, Mariska N. van Liere, Marie‐Christine M. A. B. J. Van De Glind, Marijn Muurling, Casper de Boer, Erwin G. M. Redeman, Kim A. Meijer, Wiesje M. van der Flier, Sietske A. M. Sikkes

**Affiliations:** ^1^ Alzheimer Center Amsterdam, Neurology, Vrije Universiteit Amsterdam, Amsterdam UMC location VUmc, Amsterdam Netherlands; ^2^ Amsterdam Neuroscience, Neurodegeneration, Amsterdam Netherlands; ^3^ Neurocast B.V., Amsterdam Netherlands; ^4^ Alzheimer Center Amsterdam, Neurology, Vrije Universiteit Amsterdam, Amsterdam UMC, Amsterdam Netherlands; ^5^ Faculty of Behavioural and Movement Sciences, Clinical Developmental Psychology & Clinical Neuropsychology, Vrije Universiteit Amsterdam, Amsterdam Netherlands

## Abstract

**Background:**

With the increasing number of potential interventions for Alzheimer’s Disease (AD), there is a growing need to detect meaningful cognitive changes early in the disease. Frequent passive monitoring of smartphone behaviour, such as typing speed and precision, can give insight into the cognitive changes in AD. In the ‘A personalized Medicine Approach for AD’ (ABOARD)‐project we investigated the reliability and validity of typing behaviour to monitor cognition in people with and without AD.

**Method:**

In this prospective study we included forty participants (Healthy controls (N = 9), Subjective Cognitive Decline (N = 11), Mild Cognitive Impairment (N = 10) and dementia due to AD (N = 10)). Typing behaviour and sensor data during typing were collected for 28 days using a smartphone application (Neurokeys). Eight keystroke dynamics (KD) features were calculated (Figure 1a), standardized into z‐scores, and averaged over time to create a single score per feature. Test‐retest reliability was assessed by intra‐class correlations between the first and last 14 days. Concurrent validity was explored with correlations between the Mini‐Mental State Examination (MMSE), KD features and movement in three orthogonal directions (Figure 1b).

**Results:**

All participants completed the study with an average of 640±413 keystrokes per day (Table 1). The ICC for all features range from 0.62 to 0.88, indicating good reliability. Most KD features show moderate to strong associations with MMSE performance, including the time between characters (flight time, *r* = ‐0.71, *p*<0.001), the time between the spacebar and a next character (after‐spacebar pause, *r* = ‐0.65, *p*<0.001) and the time between the backspace and a next character (post‐correction slowing, *r* = ‐0.63, *p*<0.001, Figure 2). Movement of the phone in the X (*r* = 0.39, *p*<0.05) and Z direction (*r* = 0.38, *p*<0.05) is also correlated with the MMSE (Figure 2).

**Conclusion:**

This study provides a first indication that smartphone behaviour can be a useful and reliable tool for monitoring cognition in AD. The results suggest that individuals with worse global cognition type slower, require more time to correct an error and take more time to initiate the next word, while individuals with better cognition show more movement during typing. A larger validation study can help to confirm the potential of smartphone‐derived AD digital biomarkers.